# Assessment of γ-H2AX levels in circulating tumor cells from patients receiving chemotherapy

**DOI:** 10.3389/fonc.2012.00128

**Published:** 2012-10-25

**Authors:** Alejandra Garcia-Villa, Priya Balasubramanian, Brandon L. Miller, Maryam B. Lustberg, Bhuvaneswari Ramaswamy, Jeffrey J. Chalmers

**Affiliations:** ^1^William G. Lowrie Department of Chemical and Biomolecular Engineering, The Ohio State UniversityColumbus, OH, USA; ^2^Department of Internal Medicine, Breast Medical Oncology, James Cancer Hospital and Ohio State University Comprehensive Cancer CenterColumbus, OH, USA

**Keywords:** circulating tumor cells, metastatic breast cancer, γ-H2AX, chemotherapy, Her2neu

## Abstract

Circulating tumor cells (CTCs) are prognostic markers in a variety of solid tumor malignancies. The potential of CTCs to be used as a “liquid biopsy” to monitor a patient’s condition and predict drug response and resistance is currently under investigation. Using a negative depletion, enrichment methodology, CTCs isolated from the peripheral blood of breast cancer patients with stage IV breast cancer undergoing DNA damaging therapy with platinum-based therapy were enriched. The enriched cell suspensions were stained with an optimized labeling protocol targeting: nuclei, cytokeratins 8, 18, and 19, the surface marker CD45, and the presence of the protein γ-H2AX. As a direct or indirect result of platinum therapy, double-strand break of DNA initiates phosphorylation of the histone H2AX, at serine 139; this phosphorylated form is referred to as γ-H2AX. In addition to γ-H2AX staining in specific locations with the cell nuclei, consistent with previous reports and referred to as foci, more general staining in the cell cytoplasm was also observed in some cells suggesting the potential of cell apoptosis. Our study underscores the utility and the complexity of investigating CTCs as predictive markers of response to various therapies. Additional studies are ongoing to evaluate the diverse γ-H2AX staining patterns we report here which needs to be further correlated with patient outcomes.

## INTRODUCTION

Circulating tumor cells (CTCs) have been isolated in the peripheral blood samples of patients with various kinds of solid tumor malignancies, and elevated CTC numbers correlate with adverse clinical outcomes (Pantel and Alix-Panabieres, 2007). While there is an accumulating body of evidence supporting CTCs as prognostic biomarkers, their role as predictive markers of response to various chemotherapeutic agents is currently under investigation. One such area is whether measuring changes in specific markers on CTCs during chemotherapy can select the patients who are most likely to derive benefit from a specific chemotherapy regimen. One such marker of interest is γ-H2AX.

In higher eukaryotic cells, double-strand breaks (DSBs) in DNA within chromatin quickly (within minutes) initiate the phosphorylation of the histone H2AX, at serine 139 at its C-terminus, to generate γ-H2AX. It is further noted that a single strand break of DNA can elicit a cellular mechanism that will cut the other DNA strand resulting in, effectively, a DSB. The phosphorylated form of H2AX forms foci. All cells in the body contain background foci as a result of the presence of reactive oxygen species, collapsed replication forks, eroded telomeres and depending on cell type, and growth conditions. The foci can be visualized with antibodies to γ-H2AX with each DSB yielding one focus (Nakamura et al., 2006). H2AX phosphorylation and γ-H2AX foci formation are now generally accepted as consistent and quantitative markers of DSBs, applicable even under conditions where only a few DSBs are present (Rothkamm and Lobrich, 2003).

γ-H2AX focal growth was first studied in *Muntiacus muntjak* cells. Small foci appeared 3 min after exposure to ionizing radiation (IR). The foci became brighter and larger at 9 min after IR, and reached maximal brightness and size 30 min after IR. These findings suggest that H2AX molecules in a small region near the DSB site are phosphorylated first, and are followed by molecules at increasing distances from the break site. Thus, foci are sites of accumulation for many factors involved in DNA repair and chromatin remodeling (Nakamura et al., 2006; Bonner et al., 2008; Kinner et al., 2008). There is a close correlation between γ-H2AX foci and DNA DSB numbers and between the rate of foci loss and DSB repair, providing a potentially sensitive assay to monitor DNA damage.

It is this association between γ-H2AX foci and DNA DSB numbers that has lead researchers to suggest that the detection of γ-H2AX foci in CTCs as a rapid method to assess the effectiveness of chemotherapeutic agents that induce DSB (Wang et al., 2010). Such a use of CTCs is consistent with a general term being used to describe the collection of CTCs as a “liquid biopsies”; potentially allowing near real time analysis of the action of chemotherapy agents on the patient cancer cells.

The initial study of γ-H2AX expression in CTC by Wang et al. (2010), and further studies by Kummar et al. (2011), 2012 used the FDA approved, positive selection technology for CTCs, CellSearch^TM^, for initial isolation of CTC prior to staining for γ-H2AX. While demonstrated to be effective in isolation of CTCs expressing the epithelial cells surface marker, EpCAM, significant experimental evidence is developing which suggests that potential CTCs are present in blood of cancer patients that do not express EpCAM (Sieuwerts et al., 2009; Konigsberg et al., 2011; Balasubramanian et al., 2012).

To address this bias in only selecting EpCAM positive cells, a purely negative depletion, enrichment approach was developed in which red blood cells are removed, and high expressing CD45 cells are removed in a flow through, high field magnetic quadrupole field (Lara et al., 2004; Yang et al., 2009). With this system, Jatana et al. (2010) presented results suggesting the prognostic significance of CTCs in squamous cell carcinoma of the head and neck (SCCHN) patients.

Given the initial success of this negative enrichment approach, we developed and optimized the staining protocol for evaluating γ-H2AX expression on CTCs stained using non-EpCAM positive selection which has not been previously described.

## MATERIALS AND METHODS

### CELL LINES

Two breast cancer cell lines, MCF-7 and MDA-MB-231, were procured from ATCC (Manassas, VA, USA) and grown to mid-log phase in Dulbecco’s Modified Eagle Medium (DMEM; Cat#10-013, Mediatech, Manassas, VA, USA), supplemented with 10% fetal bovine serum (FBS; Cat#30-2020, ATCC, Manassas, VA, USA) and MEM nonessential amino acids (Cat#25-025, Mediatech, Manassas, VA, USA) at 37^°^C in a humidified atmosphere containing 5% CO_2_. Cells were harvested by washing the adherent cells with PBS and subsequently incubating with Accutase^TM^ (Cat#AT104, Innovative Cell Technologies, Inc.) for 5 min at 37^°^C to remove the attached cells from the T-flask. The Accutase was then neutralized with the culture media before pelleting the cells at 350 × *g* for 5 min.

### PATIENT SAMPLES

Representative samples of CTCs from the peripheral blood of breast cancer patients with stage IV breast cancer undergoing DNA damaging therapy with platinum-based therapy were selected (OSU IRB protocol 2008C0129).

### REAGENTS USED FOR CELL SEPARATION

The blood cell suspension, after RBC lysis was labeled with tetrameric antibody complexes (TACs) from Stem Cell technologies (Cat.# 18259, Vancouver, BC, Canada). The specific TACs used in this study were a pan-leukocyte marker that targets different isoforms of the CD45 cell surface antigen and dextran coated magnetic nanoparticles.

### SEPARATION/ENRICHMENT METHODOLOGY

**FIGURE 1:**
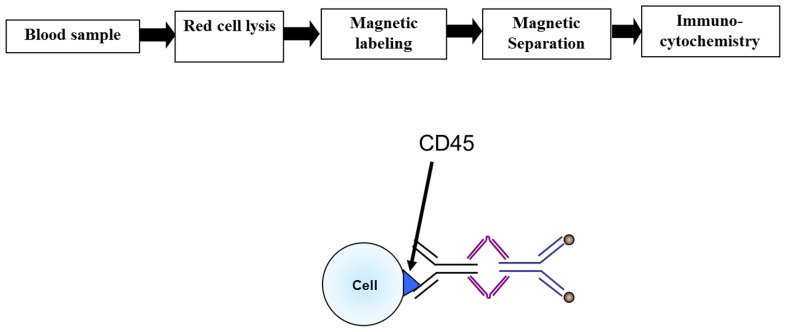
**Flow chart of the CTC enrichment methodology**.

The immunomagnetic separation was carried out as described in Yang et al. (2009) and will only be briefly summarized here. An overall view of the separation process is shown in **Figure 1**. Red blood cells in the samples were lysed by mixing the sample with lysis buffer (154 mM NH_4_Cl, 10 mM NaHCO_3_, 0.1 mM EDTA) at a ratio of 1:20, incubating it for 5 min at room temperature, and then pelleting the remaining blood cells at 350 × *g* for 5 min. This cell pellet, consisting mostly of nucleated cells, was then labeled with 0.5 μl of anti-CD45 TAC per one million cells for 30 min at room temperature on a shaker. Without washing the cells, 1 μl per one million cells of the magnetic nanoparticles were added to the cell suspension and incubated for 15 min at room temperature on a shaker. The immunomagnetically labeled cell suspension was subsequently diluted with 10 ml of buffer and run through the our previously developed, and reported, magnetic deposition system (Lara et al., 2004; Yang et al., 2009; Balasubramanian et al., 2012).

The final cell suspension, which had passed through the magnetic sorter, was then measured for volume and the cell suspension was split into two aliquots; one of which was subsequently subjected to cell lysis and RNA preservation procedures and the other either directly cytospun for analysis or preserved with 10% neutral buffered formalin for later concentration and ICC analysis. Final cell numbers were obtained by diluting 10 μl of the enriched cell suspension in 3% acetic acid (1:10 dilution) and counting the number of total cells present using a hemocytometer.

### REAGENTS, LABELING PROTOCOL, AND MICROSCOPIC IMAGING

**Table 1** lists the antibodies used for immunocytochemistry in this study and **Table 2** lists the optimized labeling protocol. An epifluorescent microscope (Nikon Eclipse 80i; Melville, NY, USA) was used for image analysis using the manufacturer supplied NIS-Elements software.

**Table 1:** Antibodies and fluoroprobes used in this study.

Target	Antibody clone	Manufacturer/Cat#	Fluoroprobe	Secondary antibody	Manufacturer/Cat#	Fluoroprobe
Nucleus	–	–	DAPI	–	–	–
Cytokeratins 8, 18, and 19	CK3-6H5	Miltenyi Biotec (130-080-101)	AF488	–	–	–
CD45	Rabbit, polyclonal	Abcam (ab10558)	–	Donkey anti-rabbit	Invitrogen (A-31573)	AF647
Phospho-histone H2A.X (Ser139)	Clone JBW301	Millipore (05-636)	–	Goat anti-mouse IgG (H + L)	Invitrogen (A-11005)	Alexa Fluor 594

**Table 2:** Optimized labeling protocol.

1. Prepare cytospin slide of enriched cell sample
2. Wash 1 × 5 min in PBST (PBS + 0.05% Tween 20)
3. Add permeabilization/blocking solution (PBS + 2% normal goat serum + 2% normal donkey serum + 1% BSA + 0.1% gelatin + 0.1% Triton X-100 + 0.05% Tween 20) for 30 min at room temperature
4. Add primary anti-CD45 (rabbit, polyclonal, 1:100) and primary anti-phospho-histone-H2A.X (Ser139; mouse, clone JBW301, 1:100) in antibody diluent, background reducing (Dako, Carpinteria, CA, USA) for 1 h at RT
5. Wash 3 × 5 min in PBST
6. Add secondary Alexa Fluor 647 donkey anti-rabbit IgG (H + L) and secondary Alexa Fluor 594 goat anti-mouse IgG (H + L) in PBST for 1 h at RT
7. Wash 2 × 5 min in PBST
8. Add custom-conjugated cytokeratin 8, 18, 19-Alexa Fluor 488 (clone CK3-6H5, 1:100) in 1% BSA/PBS for 1 h at RT
9. Wash 2 × 5 min in PBST
10. Mount with ProLong Gold antifade reagent with DAPI (Life Technologies, Grand Island, NY, USA)

## RESULTS

### POSITIVE CONTROLS: MCF-7 AND MDA-MB-213 CELLS EXPRESSING γ-H2AX FOCI

**FIGURE 2:**
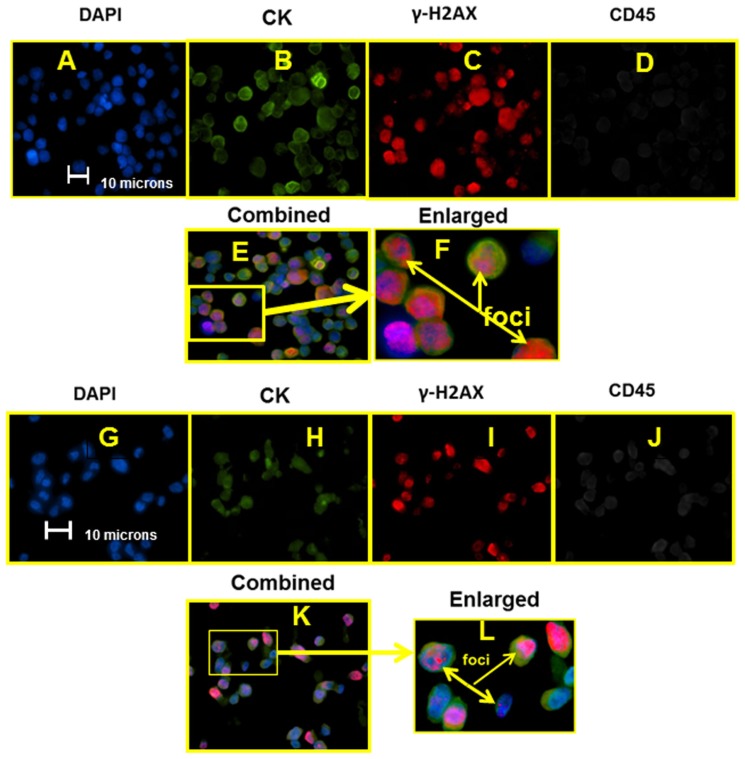
**Sets of epifluorescent images (A–F)** for MCF-7 and **(G–L)** for MDA-MB-231. For **(A–F)** and **(G–K)**, images are the same location, different filter settings corresponding to the stained marker/structure targeted and listed above the image. Figures **(F)** and **(L)** are the enlargements highlighted **(E)** and **(K)**, respectively.

**Figure 2** presents individual images, using appropriate excitation and emission, for each fluoroprobe as well as an electronically combined, and finally an electronically enlarged image of MCF-7 cells stained with the optimized labeling protocol (**Figures 2A–F**, respectively). **Figures 2G–L** presented DAPI stained and combined staining of MDA-MB-231 cell line.

### NEGATIVE CONTROLS

**FIGURE 3:**
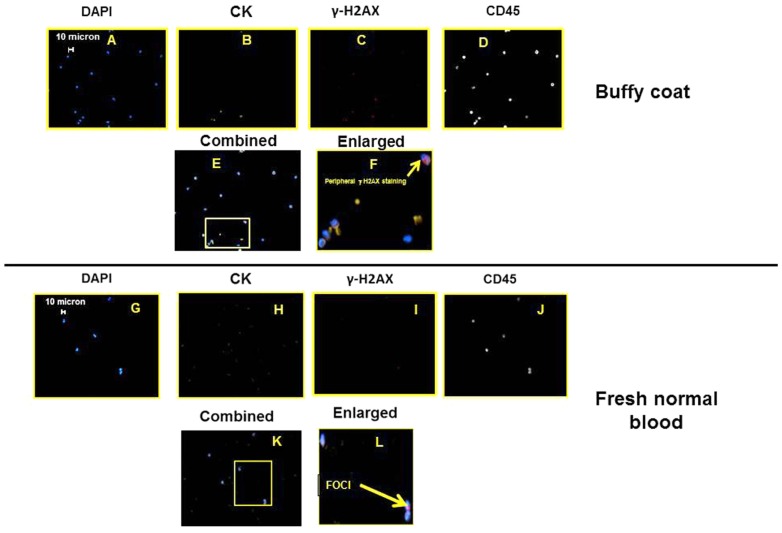
**Sets of epifluorescent images (A–F)** for buffy coat and **(G–L)** for fresh, normal blood. For **(A–F)** and **(G–K)**, images are the same location, different filter settings corresponding to the stained marker/structure targeted and listed above the image. Figures **(F)** and **(L)** are the enlargements highlighted **(E)** and **(K)**, respectively.

For a negative control, images of buffy coat and fresh, normal, human blood is presented in **Figure 3**. Note the images presented are not truly a representative image; the vast majority of cells (greater than 1 in 1000) do not stain for peripheral or foci γ-H2AX. The specific images were chosen to demonstrate that normal blood cells can have γ-H2AX staining; however, none of these γ-H2AX stained cells also co-stained for cytokeratins.

### PATIENT SAMPLES

**FIGURE 4:**
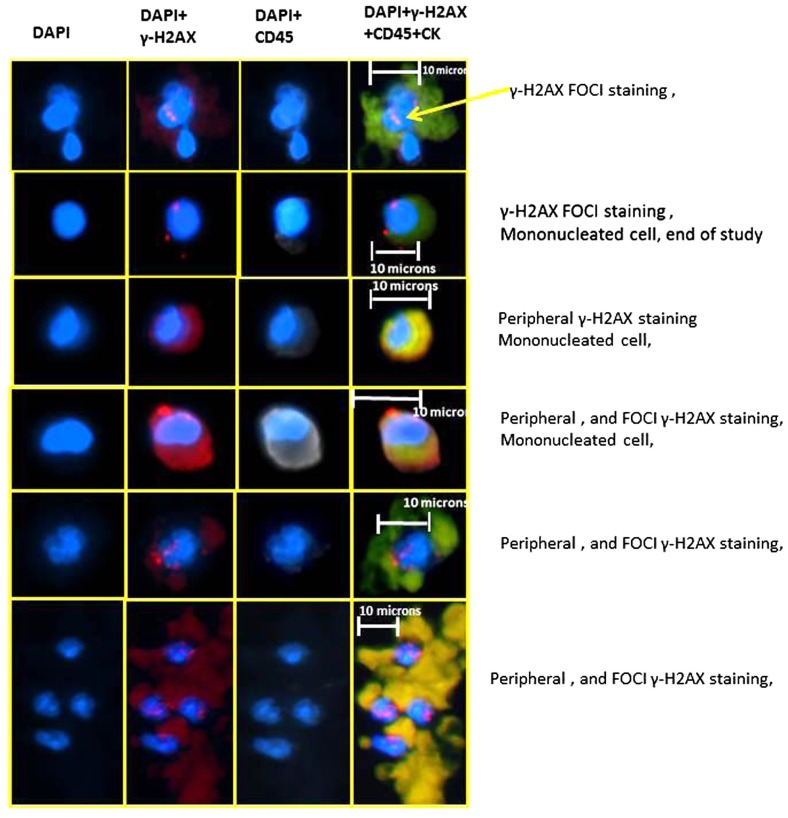
**Representative images of the different types of staining patterns observed in peripheral blood samples taken from metastatic breast cancer patients undergoing a phase I, two drug trail.** Each sample was from a different patient; yet these images represent the range of types of cells seen in a majority of patients and time points.

**Figure 4** are representative images of the different types of cell staining observed, including: (1) a traditional CTC with γ-H2AX, nuclear, foci staining, (2) a traditional CTC with only peripheral γ-H2AX staining, (3) a CTC with γ-H2AX, nuclear, foci and peripheral staining, and (4) a cohesive cluster of cells suggestive of tumor embolus that exhibits traditional CTC characteristics and γ-H2AX, nuclear, foci staining. In addition, there exists in many patient samples cells which exhibit staining for all the markers, including CD45.

## DISCUSSION

Using our optimized staining protocol, on samples obtained using an enrichment methodology which depleted normal (RBCs and CD45 expressing) cells, we successfully illustrated the presence of γ-H2AX expression on CTCs in patients with metastatic breast cancer undergoing DNA damaging chemotherapy with platinums. Our multiparameter ICC staining targeted (a) the cell nuclei, (b) cytokeratins 8, 18, and 19, (c) γ-H2AX foci, and (d) CD45. Unlike many other ICC studies of CTCs, we only used specific Alexa Fluor dyes, which have a very low rate of photo bleaching and with very low crosstalk between channels. Using this protocol, we observed various staining patterns, represented by the six different sets of images presented in **Figure 4**.

Further, the distinction between peripheral versus nuclear foci, γ-H2AX staining of cells, as well as the presence of weak CD45 staining on some of the cells, demonstrate the complexity of this type of analysis. While it has been previously reported in the literature that the occurrence of γ-H2AX staining in cells from healthy patients can occur, and as we report in **Figure 3**, we did not see in normal cells positive staining for all four markers including cytokeratin staining. In addition, in these cells from healthy patients, we did not see γ-H2AX foci staining in the nuclei. However, **Figure 4** does indicate that in addition to seeing traditional CTC (i.e., nuclei, cytokeratin positive, and CD45 negative) with γ-H2AX foci, we also observed CTCs with significant peripheral γ-H2AX staining either with or without foci staining. Thus our work suggests that there may be more than just the presence or absence of γ-H2AX foci in CTCs and this may have clinical relevance. Also, unless high magnification is used, the distinction between peripheral staining, and nuclear, foci staining, is a challenge.

Several reports exist on the presence of not just specific “foci” of staining, but the presence of an “apoptotic ring” which looks very similar to some of the staining we have seen in the enriched patient samples (Solier and Pommier, 2009). As the name implies (apoptotic ring), Solier and Pommier (2009) associate a more diffuse staining pattern of γ-H2AX in the nucleus to the apoptotic process and suggest such γ-H2AX staining as another apoptosis marker. In a separate study, Mukherjee et al. (2006) present evidence that γ-H2AX is phosphorylated during apoptotic DNA fragmentation and provide images that are similar to ones we present here.

Finally, it is possible that the peripheral staining is the result of time/aging of cells that initially had nuclear foci. Most studies of γ-H2AX staining report a rapid (within hours) appearance of foci in cancer cell lines in cell culture. Both the lack of knowledge of the pharmacodynamics in actual patients, the practicality of obtaining blood samples at regular, short intervals, and our knowledge of the half-life of CTCs in patients blood, precludes our being able at this point to determine if peripheral staining can be considered a positive response in the same manner that clear foci are considered a positive result. For example, the cells that exhibit peripheral staining in our study could have been circulating in the patient for several days, or longer, and these same cells initially could have exhibited focal staining.

Our study underscores the utility and the complexity of investigating CTCs as predictive markers of response to various therapies. Additional studies are ongoing to evaluate the diverse γ-H2AX staining patterns we report here which needs to be further correlated with patient outcomes. The use of CTCs as a “liquid biopsy” hold great promise. However, systematic, methodological studies are needed to evaluate how to develop and test unbiased multiparameter assays to test markers of interest.

## Conflict of Interest Statement

The authors declare that the research was conducted in the absence of any commercial or financial relationships that could be construed as a potential conflict of interest.
